# High-quality genome assembly of channel catfish, *Ictalurus punctatus*

**DOI:** 10.1186/s13742-016-0142-5

**Published:** 2016-08-22

**Authors:** Xiaohui Chen, Liqiang Zhong, Chao Bian, Pao Xu, Ying Qiu, Xinxin You, Shiyong Zhang, Yu Huang, Jia Li, Minghua Wang, Qin Qin, Xiaohua Zhu, Chao Peng, Alex Wong, Zhifei Zhu, Min Wang, Ruobo Gu, Junmin Xu, Qiong Shi, Wenji Bian

**Affiliations:** 1 Freshwater Fisheries Research Institute of Jiangsu Province, Nanjing, 210017 China; 2 The Jiangsu Provincial Platform for Conservation and Utilization of Agricultural Germplasm, Nanjing, 210017 China; 3 Shenzhen Key Laboratory of Marine Genomics, Guangdong Provincial Key Lab of Molecular Breeding in Marine Economic Animals, Shenzhen, 518083 China; 4 Freshwater Fisheries Research Center, Chinese Academy of Fishery Sciences, Wuxi, 214081 China; 5 BGI-Hong Kong, Hong Kong, 999077 China; 6 BGI Zhenjiang Institute of Hydrobiology, Zhenjiang, 212000 China; 7 BGI Zhenjiang Fisheries Science and Technology Industrial Co. Ltd, Zhenjiang, 212000 China; 8 Laboratory of Aquatic Genomics, College of Ecology and Evolution, School of Life Sciences, Sun Yat-Sen University, Guangzhou, 510275 China; 9 Center for Marine Research, College of Life Sciences and Oceanography, Shenzhen University, Shenzhen, 518060 China

**Keywords:** Channel catfish, Whole genome sequencing, Assembly, Gene prediction, Repetitive sequence

## Abstract

**Background:**

The channel catfish (*Ictalurus punctatus*), a species native to North America, is one of the most important commercial freshwater fish in the world, especially in the United States’ aquaculture industry. Since its introduction into China in 1984, both cultivation area and yield of this species have been dramatically increased such that China is now the leading producer of channel catfish. To aid genomic research in this species, data sets such as genetic linkage groups, long-insert libraries, physical maps, bacterial artificial clones (BAC) end sequences (BES), transcriptome assemblies, and reference genome sequences have been generated. Here, using diverse assembly methods, we provide a comparable high-quality genome assembly for a channel catfish from a breeding stock inbred in China for more than three generations, which was originally imported to China from North America.

**Findings:**

Approximately 201.6 gigabases (Gb) of genome reads were sequenced by the Illumina HiSeq 2000 platform. Subsequently, we generated high quality, cost-effective and easily assembled sequences of the channel catfish genome with a scaffold N50 of 7.2 Mb and 95.6 % completeness. We also predicted that the channel catfish genome contains 21,556 protein-coding genes and 275.3 Mb (megabase pairs) of repetitive sequences.

**Conclusions:**

We report a high-quality genome assembly of the channel catfish, which is comparable to a recent report of the “Coco” channel catfish. These generated genome data could be used as an initial platform for molecular breeding to obtain novel catfish varieties using genomic approaches.

## Data description

### Library construction, read sequencing and filtering

To generate genome sequence data, genomic DNA from mixed tissues (including muscle and skin) of channel catfish was extracted from a chosen individual cultured at a local base of the Freshwater Fisheries Research Institute (Jiangsu Province, Nanjing, China) using Qiagen GenomicTip100 (Qiagen, Hilden, DE) as per standard protocols. Isolated genomic DNA was subsequently used to construct short-insert libraries (250, 500 and 800 bp) and long-insert libraries (2, 5, 10 and 20 kb) with the standard protocol provided by Illumina (San Diego, USA). Paired-end sequencing was performed using the Illumina HiSeq 2000 platform to generate 125-bp reads using a whole genome shotgun sequencing (WGS) strategy [1].

To improve the quality of sequenced reads, we trimmed 4 bases with edges from the reads of short-insert libraries and long-insert libraries, discarded duplicated reads from the long-insert libraries, and removed reads containing 10 or more Ns and low-quality bases. Finally, a total of 201.6-Gb clean reads were generated for further genome assembly.

### Genome assembly and quality assessments

At first, we estimated the channel catfish genome size using k-mer analysis [2] with the formula: G = N*(L − 17 + 1)/K_depth, where N is the total number of reads, and K_depth indicates the frequency of reads occurring more frequently than the others. The calculated genome size is 0.839 Gb, which is shorter than that (1 Gb) from a 2016 report of an American-native channel catfish [3].

Simultaneously, we employed SOAPdenovo2 (version 2.04.4) software [4] with optimized parameters (pregraph −K 27 −d 1; contig −M 1; scaff −F −b 1.5 −p 16) to link sequenced reads to contigs and original scaffolds. All reads were then aligned onto the contigs for scaffold construction by utilizing long-insert paired-end information, which was subsequently supplied to link contigs to scaffolds in a step-wise manner. Gaps were closed using approximately 480 million of Illumina paired-end reads generated from the three libraries with insert sizes of 250, 500 and 800 bp as the input for GapCloser (v1.12-r6, default parameters and −p set to 25) [2]. A final genome assembly of 0.845 Gb in length was obtained (Table 1), which is slightly shorter than that (0.942 Gb) of a recently reported a American-native channel catfish genome [3]. The calculated contig N50 was 48.5 kilobases (kb), and the scaffold N50 was 7.2 Mb (Table 1). These values are also comparable to those in [3] (see details in Table 2).

**Table 1:** Catfish genome assembly and annotation statistics

Genome assembly	
Contig N50 size (kb)	48.5
Contig number (>100 bp)	66,332
Scaffold N50 size (Mb)	7.2
Scaffold number (>100 bp)	31,979
Total length (Mb)	845.4
Genome coverage (X)	201.6
Longest scaffold (bp)	26,612,498
Genome annotation	
Protein-coding gene number	21,556
Mean transcript length (kb)	16.1
Mean exons per gene	8.7
Mean exon length (bp)	190.2
Mean intron length (bp)	1872.4

**Table 2:** Comparison of genome assembly in sequenced fishes

Species	Sequencing platform (Mb)	Assembled genome size (Mb)	scaffold N50 (kb)	contig N50 (kb)
catfish (BGI)	Illumina	845	7248	48.5
catfish (Liu’s study [1])	Illumina, Pacbio	942	7726	77.2
zebrafish	Illumina, Sanger	1412	1551	25.0
Atlantic herring	Illumina	808	1840	21.3
greenpuffer	Sanger	342	100	16.0
medaka	Sanger	700	1410	9.8
stickleback	Sanger, Illumina	463	10,800	83.2
fugu	Sanger	332	unknown	16.5
cod	454	753	459	2.8
platyfish	454, Illumina	669	1102	21.0
lamprey	454, Illumina	816	173	unknown
lancelets	Illumina	520	unknown	unknown
tuna	454, Illumina	800	136	7.6
mudskipper	Illumina	983	2309	20.0

Two typical methods were then used to assess the quality and completeness of the generated assembly. First, transcriptome evaluation was used to assess the completeness of gene regions in the genome assembly. We carried out *de novo* assembly of the RNA sequences of skin and muscle tissues using Trinity software [5]. The assembled fragments were then aligned to the genome assembly with BLAT [6] (E-value = 10e-6, identity = 90 % and coverage >90 %). Our results indicate that the catfish genome assembly covered more than 90 % of gene-coding regions. Subsequently, Core Eukaryotic Genes Mapping Approach (CEGMA) software (version 2.3) [7] was employed with 248 conserved core eukaryotic genes (CEGs) to assess the gene space completeness within the generated genome assembly. These results demonstrate that the genome assembly covered more than 95 % of the CEG sequences, suggesting a high level of completeness.

### Transcriptome sequencing

Total RNA was extracted from muscle and skin tissues of a channel catfish (the same individual used for the above-mentioned genome sequencing) using TRIzol reagent (Invitrogen, USA). After purification using RNeasy Animal Mini Kit (Qiagen, USA), equal amounts of total RNA from each tissue were subjected to transcriptome sequencing (RNA-seq) on the HiSeq 2000 platform.

### Genome annotation

#### Repeat annotation

Firstly, RepeatModeller (version 1.04) and LTR_FINDER [8] were used to build a *de novo* repeat library with default parameters. Subsequently, RepeatMasker [9] (version 3.2.9) was utilized to map our sequences against the Repbase [10] transposable element (TE) library (version 14.04) and the *de novo* repeat library, so as to search for known and novel TEs. Next, we annotated tandem repeats using Tandem Repeat Finder [11] (version 4.04) with core parameters set as “Match = 2, Mismatch = 7, Delta = 7, PM = 80, PI = 10, Minscore = 50, and MaxPerid = 2000”. Furthermore, TE-relevant proteins were identified in our assembly using RepeatProteinMask software [9] (version 3.2.2). These identified repeat sequences accounted for 32.56 % of the channel catfish genome, of which the single largest class of TEs (representing 9.35 % of the whole genome) was the Tc1-mariner family.

#### Annotations of gene structure and function

The channel catfish genome assembly was annotated using three independent approaches: homology, *de novo* and RNA-seq annotations. For homology annotation, the protein sequences from zebrafish, Japanese fugu, spotted green pufferfish, Japanese medaka (Ensembl release 75), blue spotted mudskipper [1] and golden arowana [12] were mapped on the channel catfish genome using TblastN with e-value ≤ 1E-5. Genewise 2.2.0 software [13] was then employed to predict the potential gene structures of all alignments. Short genes (with fewer than 150 bp) and prematurely terminated or frame-shifted genes were discarded. Next, *de novo* annotation was used to annotate the gene structure from the genome assembly. We randomly selected 1000 complete genes from the homology annotation set to train the parameters for AUGUSTUS 2.5 [14]. Simultaneously, all repetitive regions were replaced in the channel catfish genome with ‘N’ to decline the ratio of pseudogene annotations. Subsequently, we utilized AUGUSTUS 2.5 and GENSCAN 1.0 [15] for *de novo* prediction of repeat-masked genome sequences. The filtered processes performed on the *de novo* annotation were the same as those used for homology prediction. Simultaneously, the RNA-seq annotation pipeline was also used to detect gene regions. We employed Tophat 1.2 software [16] to map the RNA reads extracted from the skin and muscle transcriptomes onto the channel catfish genome sequences. We then sorted and integrated Tophat alignments, and used Cufflink software [17] to analyze potential gene structures. Results from all three of the above-mentioned annotation pipelines were merged to produce a comprehensive and non-redundant gene set using GLEAN [18]. This gene set contained 21,556 genes with an average of 8.7 exons per gene (Table 1). Because different annotation pipelines were applied, the total gene number predicted here is lower than the 26,661 reported in the American-native channel catfish genome [3]. The Cuffdiff package [17] of Cufflink software (version 2.0.2.Linux_×86_64) with core parameters (−FDR 0.05 –geometric-norm TRUE –compatible-hits-norm TRUE) was utilized to calculate expression levels according to the GLEAN gene set and Tophat alignments. About 93.4 % of genes were predicted from at least two types of evidence, and approximate 78 % of the genes showed expression activity (fragments per kilobase of exon model per million mapped reads >0) in the skin and muscle tissues.

Simultaneously, all protein sequences from GLEAN results were mapped to SwissProt and TrEMBL [19] (UniProt release 2011.06) databases using BlastP [20] with an E-value ≤ 1e-5 to find the best hit for each protein. We also used InterProScan 4.7 software [21] to align the protein sequences against public databases, including Pfam [22], PRINTS [23], ProDom [24] and SMART [25], to examine the known motifs and domains in our sequences. Over 94.5 % of these predicted genes possessed at least one related functional assignment from other public databases (SwissProt [19], Interpro [21], TrEMBL and KEGG [26]). In addition, the gene structures (including exon length, intron regions and mRNAs) and exon number distributions (Table 1) were predicted to be similar to other representative teleost species such as zebrafish and medaka.

## Conclusion

We generated a channel catfish genome assembly with high quality and comparable structures to other published fish genomes, especially the Coco catfish genome [3]. This new assembly is a valuable resource and reference for further construction of high-density genetic linkage maps and identification of quantitative trait loci for molecular breeding of catfishes.

## Availability of supporting data

Supporting data are available in the GigaDB database [27]. Raw whole genome sequencing and transcriptome data are deposited in the SRA under bioproject number PRJNA319455.

## Abbreviations

BAC, bacterial artificial clone; BES, BAC end sequences/sequencing; CEG, core eukaryotic genes; CEGMA, core eukaryotic genes mapping approach; Gb, gigabases; kb, kilobases; Mb, megabases; TE, transposable element; WGS, whole genome shotgun
